# The Co-Expression and Cellular Location of HER Family Members, EGFRvIII, Putative Cancer Stem Cell Biomarkers CD44 and CD109 in Patients with Glioblastoma, and Their Impacts on Prognosis

**DOI:** 10.3390/cancers17071221

**Published:** 2025-04-04

**Authors:** Ermira Mulliqi, Said Khelwatty, Izhar Bagwan, Ahmad Kamaludin, Anna Morgan, Natalie Long, Keyoumars Ashkan, Helmout Modjtahedi

**Affiliations:** 1 School of Life Science, Pharmacy and Chemistry, Faculty of Health, Science, Social Care and Education, Kingston University London, Kingston-upon-Thames KT1 2EE, UK; k1710834@kingston.ac.uk (E.M.); s.khelwatty@kingston.ac.uk (S.K.); izhar.bagwan@nhs.net (I.B.); a.morgan@kingston.ac.uk (A.M.); 2 Berkshire Surrey Pathology Services, Royal Surrey Hospital, Guildford GU2 7XX, UK; 3 Department of Neurosurgery, Kings College Hospital, Denmark Hill, London SE5 9RS, UK; ahmad.kamaludin@nhs.net (A.K.); natalielong@nhs.net (N.L.); k.ashkan@nhs.net (K.A.)

**Keywords:** Glioblastoma, wtEGFR, EGFRvIII, HER2, HER3, HER4, CD44, CD109, prognosis

## Abstract

Background/Objectives: The aberrant expression and activation of HER family members is a known major oncogenic pathway for the proliferation, progression, and metastasis of a wide range of human malignancies. In this study, our aim was to examine the relative expression and prognostic significance of all members of the HER family, the type III EGFR mutant (EGFRvIII), and the putative stem cell markers CD44 and CD109 in patients with glioblastoma. Methods: The expression levels of wild-type EGFR (wtEGFR), HER2, HER3, HER4, EGFRvIII, CD44, and CD109 were determined in tumour specimens from 80 patients by immunohistochemistry. The staining was scored based on the percentage of positive tumour cells, the intensity, and the cellular location of immunostaining. The association between the expression level of the biomarkers and patient overall survival was evaluated using Chi-squared, Kaplan–Meier survival curves, and log-rank tests. Results: At a cut-off value of ≥5% with positive staining, 46% (wtEGFR), 75% (HER2), 19% (HER3), 71% (HER4), 85% (EGFRvIII), 95% (CD44), and 16% (CD109) of the cases were positive for these biomarkers. Interestingly, at the same cut-off value, the expression of wtEGFR in these patients was accompanied by co-expression with HER2 (35%), HER3 (0%), HER4 (30%), EGFRvIII (36%), CD44 (44%), HER2/EGFRvIII (28%), HER2/CD44 (31%), and EGFRvIII/CD44 (36%). In addition, the expression of EGFRvIII was accompanied by co-expression with HER2 (65%), HER3 (15%), HER4 (63%), CD44 (83%), CD109 (16%), wtEGFR/HER2 (28%), and 55% of the cases had co-expression of EGFRvIII/HER2/HER4/CD44. With the exception of HER2 expression, at cut-off values of ≥5% of tumour cells with positive staining, which was associated with better overall survival [HR = 0.57 (*p* = 0.038), HR = 0.56 (*p* = 0.034)], there was no significant association between the expression of other members of the HER family, EGFRvIII, CD44, and CD109 on the overall survival in both univariate and multivariate analysis. Conclusions Our results suggest that the co-expression of different members of the HER family, with EGFRvIII, CD44, and CD109, occurs in patients with glioblastoma. As the results of therapy with EGFR inhibitors have not been encouraging in patients with a brain tumour, further investigation should determine whether the co-expression of such biomarkers can be of predictive value for the response to the therapy with various types of HER inhibitors and their potential as therapeutic targets for co-targeted therapy.

## Simple Summary

Glioblastoma multiform is the most aggressive type of brain tumour. The expression of epidermal growth factor receptor (EGFR) and its mutated form EGRRvIII has been reported in patients with a brain tumour, but none of their inhibitors has been approved for the treatment of patients with a brain tumour. This study examined whether the expression of EGFRvIII is accompanied by the co-expression with other members of the HER family and putative cancer stem cell biomarkers CD44 and CD109. The results have shown that co-expression of these biomarkers occurs in patients with glioblastoma. Further investigation should determine whether the co-expression of such biomarkers can be of predictive value for the response to the therapy with various types of HER inhibitors and their potential as therapeutic targets for co-targeted therapy.

## 1. Introduction

The aberrant expression and activation of HER family members is a known major oncogenic pathway for the proliferation, progression, and metastasis of a wide range of human malignancies, including brain cancer [1,2,3]. Despite numerous efforts to target EGFR, commonly dysregulated in GBM, targeted therapeutic approaches against EGFR have not achieved the same degree of success as seen in other tumour types, particularly when compared to non-small cell lung cancer (NSCLC) [4,5]. Currently, the cure for patients with GBM has remained elusive as it requires complete destruction of the tumour by comprehensive approaches. Moreover, due to the complex and heterogeneous nature of human malignancies, including GBM, and the crosstalk between different members of the HER family and with other growth factor signalling pathways in tumour progression, the targeting of the EGFR alone may not be sufficient to produce a therapeutic effect in patients with GBM [1,6,7,8,9,10,11,12]. Therefore, it is important to discover other tumour biomarkers that are important in the progression of cancer and in mediating resistance to therapy and treatment with a combination of traditional treatment and drugs targeting such biomarkers [13,14,15,16,17]. While there have been a limited number of studies on the expression of EGFR, EGFRvIII, which is type III deletion-mutant, ligand-independent, and constitutively active form of the EGFR, there are currently no comprehensive studies of the relative expression and co-expression and prognostic significance of all members of the HER family with EGFRvIII, in patients with GBM.

Moreover, in the past few decades, the presence of cancer stem cells (CSCs), which are a minor group of cells in the tumour microenvironment, has been associated with resistance to therapeutic interventions in a wide range of cancers. This minor population of CSCs in brain tumours, which are called Brain Cancer Stem Cells (BCSS), have been associated with tumour recurrence and resistance to treatment with traditional chemotherapy and radiotherapy [18,19,20]. Of various putative BCSCS biomarkers, CD44 is a glycoprotein involved in both the migration and proliferation of cells under different oxygenation conditions [20,21,22]. Interactions between the EGFR and CD44 were reported to play a significant role in the pathology of glioblastoma. The activation of EGFR was shown to be accompanied by the upregulation of CD44 expression and the enhancement of the migratory and invasive capabilities of glioblastoma cells [22]. Moreover, the inhibition of the CD44 was found to attenuate the EGFR signalling and to enhance the sensitivity of wild-type EGFR-positive NSCLC cells to cisplatin [23]. Another factor associated with tumour progression is CD109 and the interaction of CD109 with STAT3 has been shown to drive glioblastoma stem cell plasticity and chemoresistance via STAT signalling [24]. The elevated expression of CD109 was found to promote tumour metastasis and drug resistance in lung cancer via activation of EGFR-AKT-mTOR signalling pathways and aggressiveness of cervical squamous cell carcinoma with the EGFR regulation [25,26,27,28,29]. However, there has been no study of the relative expression and prognostic significance of all members of the HER family with EGFRvIII, CD44, and CD109 in patients with glioblastoma. Therefore, in this study, for the first time, we investigated the relative expression and co-expression and prognostic significance of all members of the HER family with EGFRvIII, and cancer stem cell biomarkers CD44 and CD109 in patients with GBM.

## 2. Materials and Methods

### 2.1. Patient Information

Tumour specimens from 80 patients with brain cancer who underwent surgery, chemotherapy, or radiotherapy at Kings College Hospital, London, UK between 2017–2019 were included in this retrospective study. The ethical approval for this study was obtained by the Health Research Authority (HRA, Project IRAS ID 273040) and Kingston University Research Ethics Committee, UK. Tumour blocks in poor conditions and small biopsy samples were excluded from the study.

### 2.2. Immunohistochemical Staining

Serial sections of tumour specimens were cut from a batch of eighty embedded blocks, from patients with a brain tumour. These sections were stained using the primary antibodies, mouse anti-EGFR (DAK-H1-WT 1:100 Dako, UK), mouse anti-Her2/neu (3B5 1:200, Santa Cruz Biotechnology, Dallas, TX, USA), rabbit anti-HER3 (SP71 1:50, abcam limited, Cambridge, UK), mouse anti-HER4 (HFR1 1:100, Santa Cruz Biotechnology, Dallas, TX, USA), mouse anti-EGFRvIII (DH8.3, 1:500, Novus Bio, Leicester, UK), mouse anti-CD44 (M7982, 1:40, Agilent California, Santa Clara, CA, USA) and mouse anti-CD109 (20 µg/mL) [30]. All staining following optimisation of the antibodies was carried out on the Ultra Discovery autostainer using the ultraView DAB kit (Roche, Cornwall, UK) as described previously [29,31]. Negative sample slides (no primary antibody) were also included in the run. All slides were then counterstained with haematoxylin (0526980600, Roche, Cornwall, UK), mounted, and hand cover slipped (05424542001, Roche, UK). The entire section on the immune slide was viewed under low-power light microscopy, and the pattern of staining was noted. Then, 4 hot spot fields of staining within the tumour were selected and quantification of expression in each of the 4 fields was noted. All sections were scored depending on the percentage of tumour cells with positive immunostaining at different cut-off values (≥5%, ≥10%, ≥20%, and ≥50%), the intensity of immunostaining (i.e., 0 = negative, 1+ = weak, 2+ = moderate, and 3+ = strong) and their subcellular location (membranous, cytoplasmic, and nuclear). Two independent observers without prior knowledge of the patient’s clinicopathological features scored all slides and any disparity in scoring were resolved by simultaneous reassessment by both observers.

### 2.3. Statistical Analysis

The statistical analysis was carried out using the Statistical Package for the Social Sciences software (IBM^®^, SPSS statistics version 26, London, UK). Data are represented as mean ± SD. A *p*-value of <0.05 was considered statistically significant. The Chi-squared test (Pearson Chi-square) and Fishers’ exact test were used to assess the association between the immunohistochemistry score and the patient clinicopathological data. Kaplan–Meier survival plots were used to carry out univariate analysis and the difference between the individual groups was determined using a log-rank test. Cox-regression univariate and multivariate analysis was also conducted to confirm its significance and *p* < 0.05 was considered statistically significant and independent variable.

## 3. Results

### 3.1. Patient Clinicopathological Features

In this study, tumour specimens from 80 patients with brain cancer were examined. The median patients’ age was 62 years and ranged between 17–85 years and the median patient follow-up period was 7 years. The mean overall survival was 1.6 ± 0.21 years. The overall survival was found to be slightly poorer in males compared to females (1.36 ± 0.293 versus 1.61 ± 0.305), but such difference was not statistically significant (*p* = 0.848, Table 1). In contrast, the overall survival was significantly higher in patients who were younger than 50 years compared to those above the age of 50 (3.43 ± 0.723 versus 1.67 ± 0.147, *p* ≤ 0.1). In addition, patients with the tumour on the left hemisphere of the brain and in the parietal lobe with the presence of MGMT and IDH had reduced overall survival; however, this reduction in overall survival was not statistically significant (*p* > 0.05, Table 1).

### 3.2. The Immunohistochemical Expression of HER-Family Members and EGFRvIII in Patients with Brain Cancer

The relative expression level of all four members of the HER family (wt-EGFR, HER2, HER3, and HER4), as well as the mutant type of EGFR (EGFRvIII) proteins, were examined in the tumour specimens from these patients by immunohistochemistry. They were scored based on the percentage of tumour cells with positive immunostaining at different cut-off values, the intensity of immunostaining, and their subcellular location, as described in the previous studies with other cancers [29,31,32], and the results are summarised in Table 2. The anti-EGFR antibody used is EGFR wild type specific, and unlike other anti-EGFR antibodies used in some IHC studies, does not cross-react with the EGFRvIII deletion variant.

**Figure 1:**
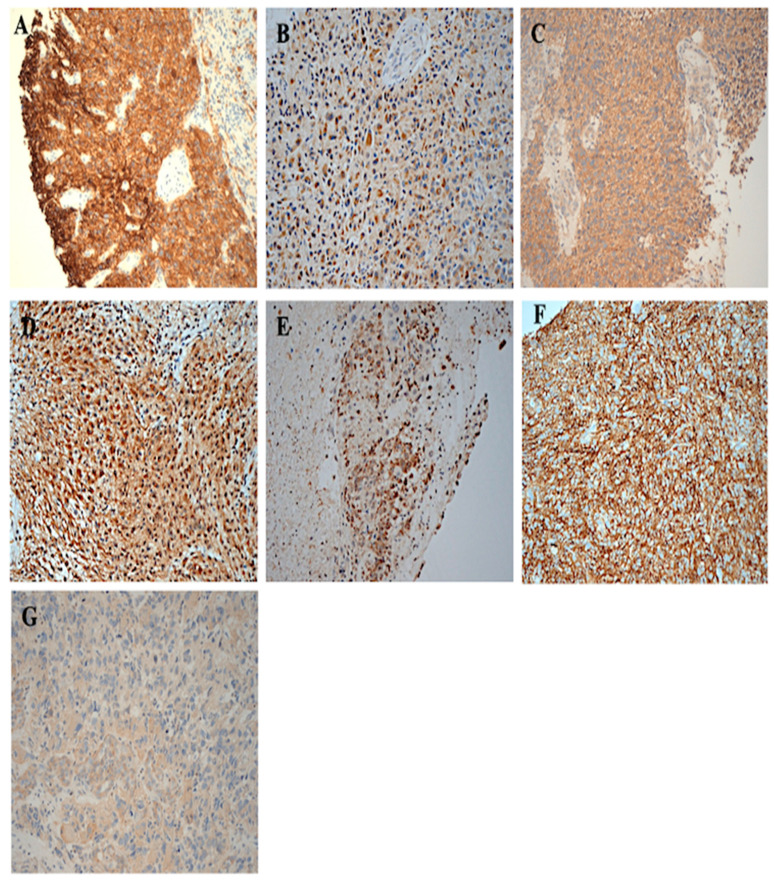
The immunohistochemical staining of whole brain tumour specimens from patients with glioblastoma for the expression of the HER-family members. Formalin-fixed paraffin-embedded tumour sections were stained with primary mAb as described under Materials and Methods. (**A**) EGFR 3+, cytoplasmic/membranous, ×200 magnification (**B**) HER2 2+ cytoplasmic, ×200 magnification (**C**) HER3 2+ cytoplasmic, ×200 magnification (**D**) HER4 2+ cytoplasmic, ×200 magnification (**E**) EGFRvIII 2+ cytoplasmic, (**F**) CD44 3+ membranous, (**G**) CD109 1+ cytoplasmic, ×200 magnification.

**Figure 2:**
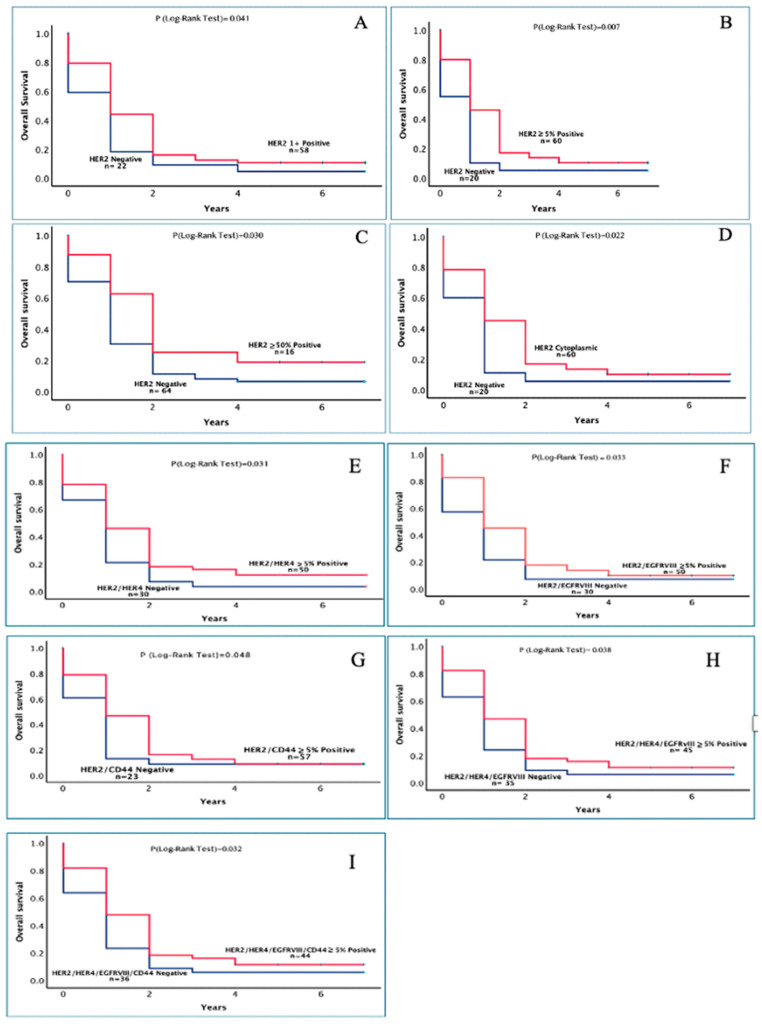
The prognostic significance in years of HER2 positive weak expression (**A**) and HER2 positive ≥ 5% (**B**) HER2 expression at ≥50% cut-off value (**C**), and HER2 cytoplasmic staining (**D**) in patients with glioblastoma. The prognostic significance in years of HER2 and its co-expression with HER4 (**E**), EGFRvIII (**F**), and CD44 at a cut-off value of ≥5% (**G**) and HER2 with HER4 EGFRvIII (**H**) HER2/HER4/EGFRvIII/CD44 at a cut-off value of ≥5% (**I**) in patients with glioblastoma. A log-rank test value of *p* < 0.05 was considered statistically significant.

At the cut-off value of ≥5% of tumour cells with immunostaining, 46% (37/80) of the cases were wt-EGFR positive, with predominant intensity and cellular location of the staining being mostly 2+ (28%) and cytoplasmic (41%) (Table 2). In 8%, and 28% of the cases examined, the intensity of staining and the cellular location of staining were strong (3+) and membranous, respectively. Interestingly, 24% of the cases had EGFR staining, which was present in ≥50% of the tumour cells (Table 2, Figure 1). At the same cut-off ≥5% of tumour cells with immunostaining, 75% of the GBM cases were HER2 positive. Of these, the predominant intensity and cellular location of HER2 staining were 1+ (418%) and cytoplasmic (75%) of the cases, respectively (Figure 2). In comparison to membranous staining of EGFR (29%), membranous expression of HER2 was present in only 5% of the cases examined (Table 2).

At the same cut-off value of ≥5% of tumour cells with positive immunostaining, 19% and 71% of the glioblastoma cases were positive for HER3 and HER4, respectively. However, the cellular location of HER3 was mainly cytoplasmic (19%). In contrast, the cellular location of HER was predominantly cytoplasmic (50%, Figure 1D), with 20% and 15% of the cases having membranous and nuclear expression of HER4, respectively (Table 2).

Next, the expression of EGFRvIII was determined, using EGFRvIII specific antibody. At the cut-off value of ≥5% of tumour cells with staining, 85% of the cases were EGFRvIII positive, with the staining intensity of 1+ (77.5%) and 2+ (10%) (Table 2). The cellular location of EGFRvIII staining was predominantly cytoplasmic (75%), and in 13% of the cases examined, the EGFRvIII was present in the nucleus (Table 2, Figure 1). Interestingly, at the cut-off value of ≥20% of tumour cells with positive staining, 41.3% of the cases were EGFRvIII, and 34%, 44%, 15%, and 56% were positive for wtEGFR, HER2, HER3, and HER4, respectively. The expression of these biomarkers at other cut-off values is presented in Table 2.

### 3.3. Immunohistochemical Expression of CD44 and CD109 in Patient with Brain Tumour

CD44 is a putative cancer stem cell biomarker and may interact with EGFR, CD109, and EGFRvIII to facilitate the tumour progression and resistance to therapies. Therefore, next the expression levels of CD44 and CD109 were also determined in the tumour specimens from these patients at different cut-off values (Table 2, Figure 1). For example, at the cut-off value of ≥5% of tumour cells with positive immunostaining, CD44 expression was detected in 95% of the cases examined, and 16% of the cases were CD109 positive. The expression of CD44 was predominantly membranous (95%), and only 1% of the patients had CD44 expression that was cytoplasmic (Table 2, Figure 1). In contrast, the intensity of CD109 staining was weak in all these patients, and the cellular location of the staining was only cytoplasmic (Table 2, Figure 1).

The co-expression of HER family members, with EGFRvIII, CD44, and CD109 was examined in the tumour specimens from patients with a brain tumour. While several studies have examined and reported the expression level of individual members of the HER family, EGFRvIII, CD44, and CD109, the number of studies on the co-expression of all members of the HER family and their co-expression with EGFRvIII, CD44, and CD109 is rare. Therefore, next and for the first time, the co-expression of these biomarkers was analyzed at different cut-off values in whole tumour brain specimens, and the results are summarized in Table 3. For example, at the same cut-off value of ≥5% of tumour cells with positive immunostaining, the expression of wtEGFR was accompanied by co-expression with HER2 (35%), HER4 (30%), EGFRvIII (36), CD44 (44%), CD109 (4%), and HER2/HER4 (29%), respectively (Table 3). Interestingly, none of the tumour specimens from the 80 cases co-expressed had co-expression of wtEGFR with HER3 or the co-expression of all four members of the HER family. At the same cut-off value, the co-expression of CD44 was present with HER2 (71.3%), HER3 (16%), HER4 (68%), EGFRvIII (83%), CD109 (16%), and EGFRvIII/CD109(16%), respectively (Table 3). The results of co-expression of HER family members with EGFRvIII, CD44, and CD109 at other cut-off values (i.e., ≥10%, ≥20%, and ≥ 50%) are summarized and presented in Table 3.

### 3.4. The Association Between the Clinicopathological Parameters and the Expression of HER-Family Members in Patients with Brain Cancer

Following the determination of the expression of HER family members, EGFRvIII, CD44, and CD109 in tumour specimens from patients with a brain tumour, a Fishers Exact correlation test was performed to find any association between the expression of these receptors and the clinicopathological features. However, there was no significant association between the expression level of the above markers and clinicopathological features of brain tumours (*p* > 0.05). 

The association between the expression level of HER-family members, EGFRvIII, CD44, and CD109, and the overall survival of Brain cancer patients. The association between the expression level of HER family members, EGFRvIII, CD44, and CD109, and the patient’s overall survival was determined using Kaplan–Meier curves and log ranks test, and the results were summarized in Table 4. With the exception of HER2 expression, at cut-off values of ≥5% of tumour cells with staining, which was associated with better overall survival [HR = 0.57 (*p* = 0.038) in univariate, HR = 0.56 (*p* = 0.034) in multivariate], there was no significant association between the expression of other members of the HER family, EGFRvIII, CD44, and CD109, at any of the cut-off values, and the overall survival in both univariate and multivariate analysis (Table 5 and Figure 2). However, the expression of HER2 at >50% or its co-expression with HER4, EGFRvIII, CD44, HER4/EGFRvIII, and HER4/EGFRvIII/CD44 at a cut-off value of ≥5% with staining were all associated with improved overall survival (Table 4 and Figure 2).

## 4. Discussion

The increased expression, mutation, and activation of the HER family members, in particular EGFR and HER2, have been reported in a wide range of human cancers and have been associated with tumour progression, resistance to therapy, and ultimately, a poorer prognosis [16,33,34,35,36,37]. More importantly, EGFR and HER2 are currently important therapeutic targets in many types of cancers, and several types of small molecule HER tyrosine kinase inhibitors or monoclonal antibodies against the EGFR and HER2 have already been approved for the treatment of patients with a wide range of human cancer [38,39,40,41,42,43]. However, to date, none of such drugs have been approved for GBM, which is the most aggressive form of brain cancer, and tumour heterogeneity may be a major contributing factor for both the primary and acquired resistance to such therapies [5,44,45,46,47,48,49,50]. Indeed, cross-talk between different members of the HER family and with CD44 or CD109 has been shown to influence tumour cell behaviour and progression [1,28,51,52]. However, to our knowledge, there has been no study on the relative expression and cellular location of all members of the HER family, EGFRvIII, and putative cancer stem cell biomarkers (e.g., CD44, CD109) and their prognostic significance when expressed alone or in combination in patients with brain tumours. Therefore, in this study for the first time, the expression level of all members of the HER family, EGFRvIII, CD44, and CD109 was determined at different cut of values in patients with glioblastoma as well as their association with the patient’s overall survival.

In this study, using anti-EGFR antibody DAK-H1-WT, which is wild-type EGFR specific and does not cross-react with EGFRvIII, the EGFR expression was determined at different cut-off values, as well as its cellular location and intensity of EGFR staining (Table 2). For example, at the cut-off value of ≥5% or ≥50% of tumour cells with positive immunostaining, 46% and 24% of the cases were wtEGFR positive (Table 2). Several studies investigated the expression level of EGFR protein in patients with brain tumour, using different antibodies, most of which were not wtEGFR specific and cross-react with EGFRvIII, and different scoring systems (Table 6). For example, in one study, Varela et al. investigated the expression of EGFR using anti-EGFR clone M3563. At a cut-off value of >10% of tumour cells with staining, they found that 22% of 103 patients were EGFR positive, with EGFR expression being significantly associated with worse overall survival [53] In another study, Chakravarti and colleagues investigated the expression of EGFR using anti-EGFR antibody Zymed (clone 31G7), which detected both the wtEGFR and EGFRvIII, in 155 tissue microarrays from patients with glioblastoma multiforme. The EGFR expression was scored by computerized quantitative image analysis, and 25% of the cases were EGFR positive, but EGFR expression was not of prognostic significance [54] (Table 6). In another study, Choi and colleagues examined the expression of EGFR in 30 patients with GBM using the same antibody 31G7, and whether the expression of EGFR influenced survival in such patients receiving postoperative radiotherapy. They found that, at a cut-off value of >10% tumour cells with staining, 77% of the cases were EGFR positive, and the EGFR positivity was associated with significantly poorer survival in both the univariate and multivariate analysis [55] In another study, again using the same antibody 31G7, Tini and colleagues investigated the EGFR expression in 144 glioblastoma patients who had undergone postoperative radiochemotherapy. They found EGFR expression in 65% of the cases examined, and high EGFR expression was associated with aggressive clinical and radiological features of GBM, leading to worse survival in such patients (74 Table 6). The expression of EGFR was determined by Hobbes and colleagues in another study using another antibody, 3C6, in 532 patients with glioblastoma. They found that 12%, 29%, and 56% of the cases had weak, moderate, and strong expression of the EGFR, respectively but the EGFR expression was not associated with overall survival in univariate analysis [56] (Table 6). In another study, Abdulghani and colleagues examined the expression of EGFR using anti-EGFR antibody EP38Y. At the cut-off value of >5% of tumour cells with staining, 39% of patients were EGFR positive, and the expression of EGFR was restricted to only grade IV glioblastoma patients, and with no expression of EGFR in the tumour from astrocytoma patients, suggesting that EGFR expression is indicative of high grades of GBM [57]. The use of different antibodies, sample sizes, patient populations, scoring methods, and brain tumour cells of various origins and tumour heterogeneity may have been some of the factors contributing to the conflicting data on the expression level of EGFR expression and their prognostic significance in patients with brain tumour. As a result, the expression of EGFR was determined in this study using a specific antibody for the wild-type EGFR with no cross-reactivity to EGFRvIII, at different cut-off values, and at different cellular locations and intensities. While tumours from these patients were wtEGFR positive, we did not find any significant associations between the expression of wtEGFR at different cut-off values and cellular location with overall survival in these patients. This highlights the need for further validation of the expression pattern and prognostic significance of wtEGFR by using larger groups of glioblastoma patients, not only based on the expression level at different cut-off values but also at different cellular locations [58,59].

**Table 6:** Studies investigating the expression level and prognostic significance of EGFR in patients with glioblastoma immunohistochemistry.

Study	Antibody Used/Scoring System	Number of Specimens Examined (% of Positive Cases)	Summary
(Agosti et al., 1992) [60]	Mouse anti-EGFR (not known).	103 astrocytic tumours, Glioblastoma (37%)	A close correlation was found between the presence of EGFR gene amplification and over-expression of receptor protein.
(Zhu et al., 1996) [61]	Mouse anti-EGFR (Sigma), EGFR positive when >0 of tumour cells with staining	55 Glioblastoma, 16 anaplastic astrocytoma (69%)	The percentage of EGFR positive cells in patients with astrocytic gliomas was associated significantly reduced overall survival (*p* = 0.0434).
(Korshunov et al., 2000) [62]	EGFR (clone H-11, Dako, catalogue No. M3563)/ ≥5% expression was considered positive	88 Ependymomas (43%)	(EGFR) revealed its predominantly membranous staining in 38 tumours with staining of 40–80% of the component cells. In addition, no differences in survival time between patients with different grades of EGFR reactivity when tumours with more than 50% of immunostained cells when compared with those with less then 50% or absence of expression.
(Andersson et al., 2004) [63]	Mouse anti-EGFR 31G7 (Zymed Laboratories)/ low (<20%), moderate (20–40%) or high (>40%)	44 Gliomas and 26 Meningiomas (58%)	The significantly higher EGFR protein expression in high-grade tumours than in low-grade tumours (*p* = 0.004) was correlated with a shorter overall survival compared to patients with low or no protein expression (*p* < 0.001). The antibody recognizes both wtEGFR and EGFRvIII.
(Nishikawa et al., 2004) [64]	EGFR 113 Novocastra (Newcastle, UK) low (<20%), moderate (20–40%) or high (>40%)	53 Glioblastoma (55%)	No prognostic significance of EGFR was studied. Also, EGFR 113 is not wt EGFR specific and cross react with the EGFRvIII
(Varela et al., 2004) [53]	EGFR (M3563, Dako Corporation, Glostrup, Denmark)/EGFR positive at >10%	103 malignant glioma (22%)	EGFR expression was significantly associated with worse overall survival (*p* < 0.01).
(Chakravarti et al., 2005) [54]	Mouse anti-EGFR 31G7, Zymed Laboratories)/ Immuno program scoring system	155 Glioblastoma multiforme TMAs (25%)	The antibody recognizes both wtEGFR and EGFRvIII. The EGFR expression was not associated with overall survival or PFS. The antibody recognizes both wtEGFR and EGFRvIII.
(Heimberger et al., 2005) [65]	Mouse anti-EGFR 31G7, Zymed Laboratories)/ >10% expression was considered positive tumour cells	196 Glioblastoma (54%)	Neither the overexpressed wild-type EGFR nor EGFRvIII was an independent predictor of median overall survival in this selected cohort of patients who underwent extensive tumour resection. The antibody recognizes both wtEGFR and EGFRvIII.
(Mendrzyk et al., 2006) [66]	EGFR (rabbit polyclonal, clone sc-03 Biotechnology, Santa Cruz, CA, USA) ≥10% expression was considered positive	163 ependymomas (59%)	EGFR overexpression was associated with a poor prognosis in patients with intracranial grade II tumours (*p* = 0.002).
(Umesh et al., 2009) [67]	EGFR (monoclonal E-30; EGFR (Clone H-11, monoclonal) A cut-off value of >20% was considered positive	54 supratentorial glioblastoma (35.2%)	Over-expression of EGFR was a significant predictor of poor outcome on multivariate analysis
(Nabika et al., 2010) [68]	Mouse anti-HER2 (Novocastra),/scored by counting the numbering of positive cells per 1000 tumour cells and producing labelling index (i.e., <30% as negative, >30% as positive).	59 High grade astrocytoma EGFR (67.8%)	High expression of EGFR was associated with poor prognosis (*p* = 0.017).
(Senetta et al., 2011) [69]	Mouse anti EGFR mAb, clone 31G7, Zymed A cut-off value of >20% was considered positive	22 supratentorial glioblastoma (45%)	By univariate analysis, histological grade (*p* = 0.018) and EGFR (*p* = 0.014) expression significantly correlated with overall survival. The antibody recognizes both wtEGFR and EGFRvIII.
(Hobbs et al., 2012) [56]	EGFR primary antibody (Ventana 790-2988/3C6/prediluted\ Sem quantitative scoring (Negative, weak, intermediate, strong)	532 glioblastomata (92%)	No significant association between EGFR expression and overall survival in univariate analysis (*p* = 0.59. The median survival was 39% longer in the high-amplifier group (*EGFR*: chromosome 7 ratio > 20) versus non-amplified GBMs (*p* = 0.03).
(Choi et al., 2013) [55]	Mouse anti-EGFR Zymed A cut-off value of >10% was considered positive	33 Glioblastoma (76.7%)	The antibody recognizes both wtEGFR and EGFRvIII. Survival in EGFR expressing GBM patients was significantly less than that in non-expressing patients (median survival: 12.5 versus 17.5 months, *p* = 0.013).
(Lee et al., 2013) [70]	EGFR, Dako, Camarillo, A cut off value of <5% was considered positive	150 Glioblastoma (62.6%)	No association between the main prognostic factors in glioblastoma.
(Michaelsen et al., 2013) [71]	EGFR DAKO, Glostrup, Denmark A cut off value of >10%, was considered positive	225 Glioblastoma multiforme (64%)	No association between the main prognostic factors in glioblastoma multiforme.
(Saha et al., 2014) [72]	Mouse anti EGFR EP38Y A cut off value of >21.7% tumour was considered positive	57 Glioblastoma (41%) Anaplastic astrocytoma’s (17%)	Distribution of age, EGFR and Ki-67 labelling index expressed strong positive (≥0.5) correlation with the grade of tumours.
(Montgomery et al., 2015) [73]	EGFR Dako, K4061 A cut off value of >25% was considered positive	36 Glioblastoma (28%)	The correlation between the expression of MDM2 and that of the wild variant of EGFR was positive (*p*-value = 0.04).
(Tini et al., 2015) [74]	Mouse anti EGFR clone 31G7, Zymed, Milan, Italy Scored based on intensity and % of tumour positively stained cells (i.e., total score 0–2 as a Low and 3–5 as a high.	144 Glioblastoma (64.6%)	The antibody recognizes both wtEGFR and EGFRvIII. Patients with a high EGFR expression seemed to present worse clinic neurological status and radiological features of tumour aggressiveness. The antibody recognizes both wtEGFR and EGFRvIII.
(Tripathy et al., 2017) [75]	Anti EGFR pan kit; Biogenex, Hyderabad, India >20% expression was considered positive	52 Glioblastoma multiforme (58%)	EGFR negative patients respond better to therapy along with longer duration of survival as compared to EGFR positive patient.
(Abdulghani et al., 2019) [57]	EGFR antibody, clone (EP38Y) Abcam >5% expression was considered positive	44 Astrocytic tumours (38.8%)	Expression of EGFR was restricted to only grade IV glioblastoma patients and no expression was found in astrocytoma patients grades I, II and III.
(Amirpour et al., 2020) [76]	EGFR antibody (Dako, Denmark) >5% expression was considered positive	70 Glioblastoma (61.4%)	GBM tumour was associated with a poor prognosis and a low survival rate. It was also found that the expression of the EGFR gene did not affect the survival rate of patients with GBM
(Miratashi Yazdi et al., 2022) [77]	Monoclonal anti-EGFR antibody clone EP22; Master Diagnóstica, Spain). >10% expression was considered positive	30 Glioblastoma (56.6%)	EGFR expression and tumour characteristics showed no significant association.
Current Study	Mouse anti-human wt EGFR mAb Clone: DAK-H1-WT Isotype: IgG1, kappa ≥5% expression was considered positive	80 Glioblastoma 46% (≥5%) 40% (≥10%) 34% (≥20%) 24% (≥50%)	No significant correlation was demonstrated between EGFR and other HER family members.

Of other members of the HER family, the expression of HER2 is more commonly associated with breast and gastric cancers, and several inhibitors have been approved for the treatment of patients with such cancers [42,78], Table 7. However, overexpression and amplification of HER2 have also been reported in a subset of glioblastoma cases in a limited number of studies (Table 7). Interestingly, of the eighty patients with glioblastoma in this study, we found that 75% of the cases expressed weak to moderate HER2 positivity, which was primarily cytoplasmic. Of these, only 5% of the patients had membranous expression of HER2 (Table 2). Moreover, HER2 expression, at cut-off values of ≥5%, was associated with better overall survival in both univariate (HR = 0.57, *p* = 0.038) and multivariate (HR = 0.56, *p* = 0.034) analysis. However, there was no significant association between overall survival and the expression of the other biomarkers in this study (Table 4 and Table 5, Figure 2). In one study, Ramezani and colleagues examined HER2 expression in 107 patients with malignant brain tumour, and of these 42% were HER2 positive. They found high expression of HER2 in high-grade gliomas supporting its potential as a therapeutic target [79]. In another study, Merurer et al. analyzed tumour specimens from 40 patients with medulloblastoma. They found HER2 positivity in 58% of the cases examined, with no significant association between HER expression and survival in such patients [80]. In another retrospective study, Mineo and colleagues examined HER2 expression in 57 patients with GBM and found that 83% of GBM cases were moderately to highly positive for HER2 expression. They found GBM with low HER2 expression to be more likely from secondary GBM and with a better prognosis than de novo GBM [81]. Interestingly, in our study, we also found that the survival time in patients with low expression of HER2 was significantly longer in such patients, which could be due to the mainly cytoplasmic expression of HER in our study (Table 4, Figure 2). Therefore, it is important to determine the relative expression and cellular location of HER2 in a larger group of GBM patients and its potential as a therapeutic target, prognostic marker, and predictive indicator for the response to therapy with the EGFR and HER inhibitors.

Of the HER family members, at the cut-off value of >5% of tumour cells with staining, we found that 19% and 71% of the cases were found to be HER3 and HER4 positive. However, there was no statistically significant association between the expression level of HER3 and HER4 and the overall survival of glioblastoma patients in this present study (*p* > 0.05). At present, there are a limited number of studies on the expression level, cellular location, and prognostic significance of HER3 and HER4 in patients with brain cancer in the literature (Table 7). In one study, Nabika and colleagues examined the expression of HER3 and HER4 in 59 patients with high-grade astrocytoma. They found HER3 and HER4 overexpression in 5% and 75% of the cases examined, respectively, and of these, only HER4 overexpression was associated with poor prognosis [68]. In another study, Arnli and colleagues determined the expression levels of HER3 and HER4 in the TMAs from 186 patients with meningiomas. At a cut-off value of >10% of tumour cells with staining, they found HER3 and HER4 expression in 99% and 100% of the cases examined (Table 7). However, they found no significant association between Correctedthe expression of HER3 or HER4 and overall survival in their patients. In this study, at the same cut of the value of >10% of staining, we found a lower percentage of the patients were HER3 (17%) and HER4 (67%) positive (Table 2 and Table 7). However, like other studies, we did not find any significant association between the expression of HER3 and overall survival. Interestingly, in another study, Kusuhara and colleagues investigated the membranous expression of HER2 and HER3 in 44 patients with brain metastasis of the breast. They found the tissue that, unlike HER2, had little change in the immunohistochemistry score between primary and metastatic brain tissues, breast cancer brain metastases had significantly higher levels of HER3 expression (91%) than primary tumours (59%), concluding that HER3 could be a potential therapeutic target in patients with brain metastasis [82]. In another study, HER3 expression was determined in 180 patients with metastatic breast cancer and NSCLC cancer in the brain. HER3 expression was found in 75% of brain metastasis, supporting its potential as a target therapy for brain cancer metastasis [82,83,84,85,86].

**Table 7:** Studies investigating the expression level and prognostic significance of HER2, HER3 and HER4 in patients with glioblastoma immunohistochemistry.

Study	Antibody Used/Scoring System	Number of Specimens Examined (% of Positive Cases)	Summary
(A) HER2			
(Haapasalo et al., 1996) [87]	Anti HER2 monoclonal MAb1 antibody TA250	94 Glioblastoma (6.4%)	No significant prognostic value and that HER2 oncogene amplification is not seen in the few cases in which there is overexpression.
(Koka et al., 2003) [88]	HER2 (DAKO Diagnostics)	149 Glioblastoma 15.4%	Age, performance status, smoking history, and treatment, logistic regression analysis (with a survival of <3 months as the dependent variable) revealed that Her-2/neu overexpression significantly (*p* < 0.01) increased the odds of early mortality (<3 months).
(Mineo et al., 2007) [81]	Anti-HER2 antibody (Novocastra, clone CB11) 0 = no staining; 1+ = faint, incomplete membranous pattern; 2+ = moderate, complete membranous pattern; 3+ = strong membranous pattern	57 Glioblastoma (82.5%)	Survival time was significantly longer when HER2 expression was low *p* = 0.04). The patterns of HER2 expression were similar between grade III gliomas and secondary GBM.
(Meurer et al., 2008) [80]	Mouse anti-HER2, Neu F-11 (Santa Cruz Biotechnology) >20% expression was considered positive tumour cells	40 Medulloblastoma (57.5%)	HER2 was positive in 23 (57.5%) of the samples and did not show statistical association with survival (*p* = 0.07).
(Nabika et al., 2010) [68]	Mouse anti-HER2 (Novocastra),/scored by counting the numbering of positive cells per 1000 tumour cells and producing labelling index (i.e., < 30% as negative, >30% as positive).	59 High grade astrocytoma HER2 (28.8%)	No significant association between HER2 overexpression and prognosis
(Ramezani et al., 2020) [79]	HER2 (DAKO Diagnostics, Polyclonal Rabbit Anti-Human A0485) >10% expression was considered positive tumour cells	107 Malignant brain tumours (42.1%)	The type of brain tumours can impact on HER2 expression that high HER2 expression in High grade glioma may be helpful for therapeutics.
Mulliqi et al., 2025 Current study	Mouse anti-human HER-2 mAb Clone: 3B5 Isotype: IgG1, kappa ≥5% expression was considered positive tumour cells	80 Glioblastoma 75% (≥5%) 55% (≥10%) 43.8% (≥20%) 20.% (≥50%)	Co expressions with low HER2 intensity there was a statistically significant association on increase of patient overall survival. HER2 positivity was found to be an independent prognostic marker in multivariate analysis. Furthermore, expression of HER2 positivity with cytoplasmic staining was also associated with significantly increase of overall survival in patients with glioblastoma (*p* = 0.022).
(B) HER3 and HER4			
(Nabika et al., 2010) [68]	Mouse anti-HER3 (Novocastra), and HER4 (Lab vision) antibodies/scored by counting the numbering of positive cells per 1000 tumour cells and producing labelling index (i.e., < 30% as negative, >30% as positive).	59 High grade astrocytoma HER3 (5.1%) HER4 (75%)	High expression of HER4 was associated with a poor prognosis (*p* = 0.004) but no association between HER3 and prognosis
(Donoghue et al., 2018) [89]	Anti HER4 antibody (not known) <50% as negative and >50% as positive	53 Glioblastoma HER4 (11%)	high p-ERBB4 in 11% of archived GBM samples, independent of p-EGFR, was associated with shorter patient survival (12.0 ± 3.2 months) than was no p-ERBB4 (22.5 ± 9.5 months)
(Arnli et al., 2019) [90]	HER3 RTJ-1 IgM, mouse monoclonal HER4 HFR1 IgG2b, mouse Monoclonal, Dako >10% expression was considered positive	186 Meningiomas TMA HER3 (98.4%) HER4 (100%)	Meningiomas of all grades were shown to widely express both HER3 and HER4 receptors however neither HER3 nor HER4 expressions to be of prognostic significance.
(Kusuhara et al., 2022) [82]	HER3 (Cell Signalling Technology) H score system	44 Metastatic brain cancer HER3 (91%)	Tissue from breast cancer brain metastases had significantly higher levels of HER3 expression than primary tumours, supporting that HER3 is a potential target for BC brain metastases.
(Tomasich et al., 2023) [85]	HER3 (Cell Signalling Technology, 12708, RRID: AB_2721919 >10% expression was considered positive	Metastatic brain cancer HER3 180 (75%)	HER3 expression did not correlate with overall survival from Brain metastasis diagnosis.
Mulliqi et al., 2025 Current study	Rabbit anti-HER3 mAb Clone: SP71 Isotype: IgG Mouse anti-human HER4 mAb Clone: HFR1 Isotype: IgG2b, kappa ≥5% expression was considered positive	80 Glioblastoma 19% HER3 (≥5%) 18%HER3(≥10%) 15% HER3 (≥20%) 4% HER3(≥50%) 71%HER4 (≥5%) 68%HER4(≥10%) 56%HER4(≥20%) 25% HER4(≥50%)	HER3 expression was found not to be of prognostic significance. There was found to be a prognostic significance in years of HER2 and its co expression with HER4 on the overall survival.

In addition to overexpression of EGFR, in several studies, mutations in the intracellular and extracellular domains of the EGFR have been associated with tumour progression. In this study, 85% of patients with glioblastoma were found to be EGFRvIII positive with weak to moderate staining (Table 2). EGFRvIII has been identified for the first time in patients with a brain tumour and has been extensively studied for its role in tumour progression, cancer prognosis, and therapeutic targets in the past four decades [91,92,93,94,95]. The reported expression of EGFRvIII in patients with glioblastomas varies from 19% to 57% in the literature (Table 8). For example, in one study involving 67 glioblastoma patients, Nozawa and colleagues found 19% of the cases were EGFRvIII. Pelloski and colleagues examined EGFRvIII in 649 patients with glioblastoma, and at a cut-off value of >10% of tumour with staining, and 31% of the cases were EGFRvIII positive. However, they found no significant association between overall survival and positivity for EGFRvIII [96]. Felsburg and colleagues examined tumour specimens from 106 patients with glioblastoma and found 57% of the cases to be EGFRvIII positive. However, like our study, they did not find any associations between EGFRvIII positivity and overall survival in patients with glioblastoma (Table 8) [15]. Therefore, taken together, these results suggest that expression of EGFRvIII occurs in patients with GBM and that this biomarker may be a good candidate for targeted therapy with various types of HER inhibitors, including anti-EGFR, anti-EGFRvIII antibodies, the EGFRvIII T cell bispecific antibody, the anti-EGFRvIII antibody–drug conjugate, and CAR-T cells [94,97,98,99].

**Table 8:** Studies investigating the expression level and prognostic significance of EGFRvIII in patients with Glioblastoma by immunohistochemistry.

Study	Antibody Used/Scoring System	Number of Specimens Examined (% of Positive Cases)	Summary
(Shinojima et al., 2003) [100]	EGFRvIII DH8.3 no staining), 1(light or focal), 2 (moderate), and 3 (strong).	87 supratentorial glioblastoma (45%)	EGFRvIII overexpression, was not predictive for OS. However, in patients with EGFR amplification, multivariate analysis revealed that EGFRvIII overexpression was an independent, significant, poor prognostic factor for OS (*p* = 0.0044, HR = 2.71).
(Aldape et al., 2004) [101]	Rabbit anti EGFRvIII Zymed, San Francisco, CA, USA >10% expression was considered positive	168 Glioblastoma (41.3%) Anaplastic astrocytoma (21.4%)	EGFRvIII had positivity but no association with overall survival among GBM patients (*p* = 0.84) but being highly associated with reduced survival among Anaplastic astrocytoma (AA) patients (*p* = 0.0016). The antibody recognizes both wtEGFR and EGFRvIII.
(Heimberger et al., 2005) [65]	Rabbit anti-EGFRvIII polyclonal antibody (Zymed, San Francisco, CA, USA). >10% expression was considered positive	196 Glioblastoma multiforme (31%)	Neither the overexpressed wild-type EGFR nor EGFRvIII was an independent predictor of median overall survival. The antibody recognizes both wtEGFR and EGFRvIII.
(Pelloski et al., 2007) [96]	EGFRvIII Zymed, Carlsbad, CA, USA) >10% expression was considered positive	649 Glioblastoma (31%)	No significant association between shorter overall survival time and positivity for EGFRvIII (*p* 0.056) and p-Akt (*p* 0.095) and extent of resection (*p* 0.091).
(Felsberg et al., 2017) [15]	EGFRvIII monoclonal mouse antibody E30 (Dako) Semiquantitative scored	106 Glioblastoma (56.6%)	EGFRvIII positivity was not associated with different progression-free or overall survival
(Nozawa et al., 2019) [102]	EGFRvIII (clone L8A4; Absolute Antibody, Oxford, UK. >30% expression was considered positive	67 Glioblastoma (19.4%)	EGFRvIII-expression in patients with glioblastoma was not significantly associated with a favorable outcome.
Mulliqi et al., 2025 Current study	Mouse anti-EGFRvIII mAb. Clone: DH8.5 (IgG1) ≥5% expression was considered positive	80 Glioblastoma 85% (≥5%) 59% (≥10%) 41% (≥20%) 4% (≥50%)	EGFRvIII had no prognostic significance.

As mentioned above, CD44 and CD109 are both cell surface glycoproteins that play significant roles in cancer biology [22,103]. The expression of both CD44 and CD109, which act as putative cancer stem cell biomarkers, facilitate cancer progression and resistance to treatment, making them promising targets for therapeutic strategies in various cancers [27,52,104]. The overexpression of CD44 has been reported in many studies but not so much for CD109 in glioblastoma patients. In this study, 95% of the cases examined had membranous expression of CD44. In agreement with the present study, Si et al. have recently shown that CD44 in GBM tissues is mainly expressed on the cell membrane but in contrast to this study, they found high levels of CD44 expression to be associated with a poorer prognosis [105]. Many studies have reported that high expression CD44 predicts poor prognosis in other cancer types [106,107,108]. Although CD44 expression is recognized as a definite independent prognostic factor in various cancers, CD44 isoforms have increasingly attracted attention due to their much more specific expression in cancer stem cells and their various functions via signalling transduction compared with CD44 [22]. On the other hand, CD109 overexpression has been reported in some cancers and CD109 expression has been associated with chemoresistance by upregulation of EGFR, and alteration of the tumour microenvironment [27,29,30,109,110]. To date, there are limited studies of CD109 in patients with a brain tumour. In one study, the high expression of CD109 expression has been correlated with increased activity of the STAT3 pathways and causing high proliferation of glioblastoma cells [24]. In another study, CD109 expression was found to promote the progression of glioma and tumour metastasis and resistance via the EGFR pathway expression [25,26]. Moreover, another study has shown that patient-derived CD109 positive cells are highly enriched with clonogenic, tumour-initiating, and radiation-resistant properties, and the silencing of CD109 resulted in the significant inhibition of these phenotypes [111]. In this study, at cut-off values ≥5% and >50%, only 16% and 8% of the GBM cases were CD109 positive. Therefore, further research is needed to understand the exact mechanisms by which CD109 expression and its co-expression with other biomarkers contributing to tumour progression in glioblastoma.

In recent years, the interplay between members of the HER family receptors with EGFRvIII and cancer stem cell biomarkers like CD44 and CD109 have been associated with the aggressive nature of glioblastoma [22,112,113]. In this study for the first time, we found the co-expression of wtEGFR with HER2 (35%), HER4 (30%), EGFRvIII (36%), CD44 (44%), and CD109 (3.9%) (Table 3). However, none of the patients had co-expression of the wtEGFR with HER3 nor the co-expression of all the four members of the HER family (Table 3). Moreover, at cut-off value ≥5% tumour cells with positive immunostaining, the expression CD44 was found to be accompanied by the co-expression with individual members of the HER family as well as EGFRvIII (83%), CD109 (16%), HER2/EGFRvIII (65%), or HER2/HER4/EGFRvIII (55%) (Table 3). However, there was no significant association between the expression of these biomarkers and overall survival in both univariate and multivariate analyses (Table 5). Interestingly, in Kaplan–Meier survival analysis, at cut-off value ≥5% of tumour cells with staining, only the co-expression of HER2/HER4 was associated with statistically significant prolonged survival (1.08 ± 0.25 vs. 1.94 ± 0.29, *p* = 0.031). However, it did not remain an independent prognostic factor for poor overall survival in both univariant and multivariant analysis (Table 5, Figure 2). In agreement with the present study, Bodey et al. found that tumours from 11/22 patients with medulloblastoma tumours had co-expression of HER2 with HER4 and this was highly detectable in only high-grade glial tumours [114]. Interestingly, in a recent study, using the same reagents and scoring system, Al-Janaby and colleagues examined the co-expression of HER family members and CD44 in 78 patients with stomach cancer. Of these, the co-expression of HER2/EGFRvIII, EGFRvIII/CD44, and EGFRvIII/CD44/HER2 were all associated with poorer overall survival [115] Tina Al-Janaby et al., 2025, The co-expression and prognostic significance of the HER-family members, EGFRvIII, CD44, CD109, and Claudin 18.2 in patients with adenocarcinoma, 3341 (in press)). The heterogeneous expression of HER family members and putative cancer stem cell biomarkers suggests the complex regulatory network that may drive brain tumour progression and its resistance to therapy with various types of EGFR inhibitors [14,49,116,117,118] Therefore, further investigations involving a larger group of patients are warranted to validate the relative expression, co-expression, and prognostic significance of all members of the HER family with EGFRvIII and CD44 in patients with brain tumours.

## 5. Conclusions

In summary, here, we provided the first comprehensive study of the co-expressions, cellular location, and prognostic significance of all members of the HER family and EGFRvIII in patients with brain cancer. Taken together, our results suggest that the co-expression of different members of the HER family, with EGFRvIII, CD44, and CD109 is common in patients with glioblastoma. Further investigations are warranted to determine their potential as targets for therapeutic interventions with drugs co-targeting these receptors and their predictive values for the response to therapy with the HER inhibitors in patients with GBM.

## Figures and Tables

**Table 1:** Patient clinicopathological features and their association with overall survival using Kaplan–Meier analysis and log-rank test in eighty glioblastoma tumour specimens.

Characteristics	Number of Patients (%)	Overall Survival in Years (Mean ± SE)	95% CI	*p*-Value
Age				
<50	16 (20)	3.43 ± 0.72	2.02–4.85	<0.1
≥50	64 (80)	1.67 ± 0.15	0.89–1.45	
Gender				
Male	49 (61.3)	1.36 ± 0.29	1.06–2.21	0.848
Female	31 (38.8)	1.61 ± 0.31	1.02–2.21	
Location				
Frontal	17 (21.3)	1.18 ± 0.32	0.54–1.81	0.068
Parietal	19 (23.8)	2.70 ± 0.65	1.42–3.96	
Other	44 (55)	1.30 ± 0.92	0.92–1.67	
Hemisphere				
Left	39 (48.8)	1.54 ± 0.30	0.96–2.12	0.191
Right	37 (46.3)	1.83 ± 0.30	1.19–2.48	
Both	4 (5.0)	0.50 ± 0.50	0–1.48	
O6-methylguanine-DNA methyl-transferase (MGMT)				
Present	41 (51.2)	1.94 ± 0.32	1.33–2.56	0.066
Absent	39 (48.8)	1.31 ± 0.28	0.76–1.85	
Isocitrate dehydrogenase (IDH)				
Present	2 (2.5)	3.00 ± 0.72	1.16–4.83	0.091
Absent	78 (97.5)	1.153 ± 0.208	0.208–1.14	

**Table 2:** The expression of HER-family members, EGFRvIII, and cancer stem cell markers CD44 and CD109 was determined by immunohistochemistry in tumour specimens from 80 patients with brain cancer.

Scoring Criteria	Wt-EGFR	HER2	HER3	HER4	EGFRvIII	CD44	CD109
Cut-off value Number of positive (%)							
≥5%	37 (46)	60 (75)	15 (19)	57 (71)	68 (85)	76 (95)	13 (16)
≥10%	32 (40)	44 (55)	14 (18)	54 (68)	47 (59)	72 (90)	13 (16)
≥20%	27 (34)	35 (44)	12 (15)	45 (56)	33 (41)	66 (83)	12 (15)
≥50%	19 (24)	16 (20)	3 (4)	20 (25)	3 (4)	33 (41)	6 (8)
Intensity							
1+	12 (15)	58 (41)	15 (19)	43 (54)	62 (78)	53 (66)	13 (16)
2+	22 (27.5)	13 (9)	1 (1)	18 (23)	8 (10)	45 (56)	0 (0)
3+	11 (8)	0 (0)	0 (0)	0 (0)	0 (0)	13 (16)	0 (0)
Sub-cellular localization							
Membranous	23 (29)	4 (5)	0 (0)	16 (20)	0 (0)	76 (95)	0 (0)
Cytoplasmic	33 (41)	60 (75)	15 (18)	40 (50)	60 (75)	1 (1)	13 (16)
Nuclear	1 (1)	0 (0)	0 (0)	4 (15)	10 (13)	0 (0)	1 (1.3)

**Table 3:** Co-expression of HER family members, EGFRvIII, CD44, and CD109 in brain cancer tumour samples at different cut-off values.

Markers	Number of Positive Tumours (%)			
	≥5% Cut Off	≥10% Cut Off	≥20% Cut Off	≥50% Cut Off
wtEGFR/HER2	28 (35)	18 (23)	10 (13)	5 (6)
wtEGFR/HER3	0 (0)	0 (0)	0 (0)	0 (0)
wtEGFR/HER4	24 (30)	24 (30)	14 (18)	4 (5)
WtEGFR/EGFRvIII	29 (36)	14 (18)	12 (15)	0 (0)
wtEGFR/CD44	35 (44)	30 (38)	25 (31)	10 (13)
wtEGFR/CD109	3 (4)	2 (3)	1 (1)	1 (1)
wtEGFR/HER2/HER3	0 (0)	0 (0)	0 (0)	0 (0)
wtEGFR/HER2/HER4	23 (29)	16 (20)	8 (10)	2 (3)
WtEGFR/HER3/HER4	0 (0)	0 (0)	0 (0)	0 (0)
wtEGFR/HER2/EGFRvIII	22 (28)	11 (14)	5 (6)	0 (0)
wtEGFR/HER2/CD44	25 (31)	17 (21)	10 (13)	2 (3)
wtEGFR/HER2/CD109	3 (4)	0 (0)	0 (0)	0 (0)
wtEGFR/HER3/EGFRvIII	0 (0)	0 (0)	0 (0)	0 (0)
wtEGFR/HER3/CD44	0 (0)	0 (0)	0 (0)	0 (0)
wtEGFR/HER3/CD109	0 (0)	0 (0)	0 (0)	0 (0)
wtEGFR/HER4/EGFRvIII	19 (24)	11 (14)	5 (6)	0 (0)
wtEGFR/EGFRvIII/CD44	29 (36)	16 (20)	12 (15)	0 (0)
wtEGFR/EGFRvIII/CD109	4 (5)	2 (3)	0 (0)	0 (0)
wtEGFR/HER2/HER3/HER4	0 (0)	0 (0)	0 (0)	0 (0)
HER2/HER3	13 (16)	11 (14)	7 (9)	1 (1)
HER2/HER4	50 (63)	35 (44)	24 (30)	6 (8)
HER2/EGFRvIII	52 (65)	29 (36)	17 (21)	0 (0)
HER2/CD44	57 (71)	41 (51)	29 (36)	6 (8)
HER2/CD109	11 (14)	9 (11)	6 (8)	2 (3)
HER2/HER3/HER4	12 (15)	10 (13)	5 (6)	0 (0)
HER2/HER3/EGFRvIII	11 (14)	9 (11)	4 (5)	0 (0)
HER2/HER3/CD44	14 (85)	11 (14)	7 (9)	0 (0)
HER2/HER3/CD109	5 (6)	4 (5)	3 (4)	0 (0)
HER2/HER4/EGFRvIII	45 (56)	21 (26)	11 (14)	0 (0)
HER2/HER4/CD44	48 (60)	33 (41)	19 (24)	3 (4)
HER2/EGFRvIII/CD44	52 (65)	27 (34)	15 (19)	0 (0)
HER2/HER4/CD109	11 (14)	8 (10)	2 (3)	0 (0)
HER2/EGFRvIII/CD109	12 (15)	7 (9)	5 (6)	0 (0)
HER2/CD44/CD109	12 (15)	10 (13)	8 (10)	0 (0)
HER2/HER4/EGFRVIII/CD44	44 (55)	19 (24)	8 (10)	0 (0)
HER3/HER4	13 (16)	12 (15)	9 (1)	0 (0)
HER3/EGFRvIII	12 (15)	11 (14)	6 (8)	0 (0)
HER3/CD44	13 (16)	14 (18)	12 (15)	1 (1)
HER3/CD109	4 (5)	4 (5)	3 (4)	0 (0)
HER3/HER4/EGFRVIII	12 (15)	10 (13)	6 (8)	0 (0)
HER3/HER4/CD109	4 (5)	4 (5)	2 (2)	0 (0)
HER3/HER4/CD44	13 (16)	12 (15)	9 (11)	0 (0)
HER3/EGFRvIII/CD44	13 (16)	11 (14)	9 (11)	0 (0)
HER3/EGFRVIII/CD109	5 (6)	5 (6)	3 (4)	0 (0)
HER3/CD44/CD109	5 (63)	5 (6)	3 (4)	0 (0)
HER4/EGFRvIII	50 (63)	31 (39)	17 (21)	1 (1)
HER4/CD44	54 (68)	49 (61)	35 (44)	8 (10)
HER4/CD109	12 (15)	8 (10)	8 (10)	1 (1)
HER4/EGFRvIII/CD44	48 (60)	29 (36)	14 (18)	0 (0)
HER4/EGFRvIII/CD109	11 (14)	8 (10)	4 (5)	0 (0)
HER4/CD44/CD109	12 (15)	10 (13)	7 (9)	0 (0)
EGFRvIII/CD44	66 (83)	44 (55)	29 (36)	1 (1)
EGFRvIII/CD109	13 (16)	11 (14)	5 (6)	0 (0)
EGFRvIII/CD44/CD109	13 (16)	11 (14)	4 (5)	0 (0)
CD44/CD109	13 (16)	13 (16)	11 (14)	0 (0)

**Table 4:** The association between the sub-categories of receptor expression and the overall survival of brain cancer patients. Overall survival was determined by Kaplan–Meier curves and the log-rank test. A *p*-value of <0.05 was considered significant.

	Overall Survival (Years)		
Receptor Expression	Negative	Positive	*p*-Value
HER2 Intensity 1+	1.09 ± 0.34	1.83 ± 0.26	0.041
HER2 ≥ 5%	0.90 ± 0.33	1.87 ± 0.25	0.7
HER2 ≥ 50%	1.39 ± 0.21	2.56 ± 0.57	0.030
HER2 Cytoplasmic	0.98 ± 0.35	1.83 ± 0.25	0.022
HER2/HER4 ≥ 5%	1.08 ± 0.25	1.94 ± 0.29	0.031
HER2/EGFRVIII ≥ 5%	1.43 ± 0.33	1.89 + 0.27	0.033
HER2/CD44 ≥ 5%	1.17 ± 0.39	1.81 ± 0.25	0.048
HER2/HER4/EGFRVIII ≥ 5%	1.19 ± 0.28	1.95 ± 0.30	0.038
HER2/HER4/EGFRVIII/CD44 ≥ 5%	1.19 ± 0.27	1.97 ± 0.27	0.032

**Table 5:** Univariate and multivariate analysis of the association between sub-categories of various receptors and overall survival. A *p*-value of <0.05 was considered significant. Overall survival relative to the indicated features was determined by Cox regression analysis.

Expression in Years	Univariate			Multivariate		
	Hazard Ratio	95% CI	*p*-Value	Hazard Ratio	95% CI	*p*-Value
HER2 Intensity 1+	0.665	0.398–1.111	0.119	0.677	0.403–1.137	0.140
HER2 ≥ 5%	0.567	0.331–0.970	0.038	0.556	0.323–0.958	0.034
HER2 ≥ 50%	0.608	0.332–1.114	0.107	1.37	0.910–2.081	0.130
HER2 Cytoplasmic	0.612	0.354–1.056	0.078	0.650	0.373–1.132	0.128
HER2/HER4 ≥ 5%	0.669	0.414–1.083	0.102	0.706	0.433–1.151	0.162
HER2/EGFRVIII ≥ 5%	0.670	0.413–1.089	0.106	0.710	0.435–1.158	0.170
HER2/CD44 ≥ 5%	0.669	0.398–1.122	0.128	0.687	0.405–1.164	0.163
HER2/HER4/EGFRVIII ≥ 5%	0.686	0.686–1.098	0.116	0.718	0.445–1.156	0.173
HER2/HER4/EGFRVIII/CD44 ≥ 5%	0.678	0.424–1.085	0.105	0.702	0.436–1.133	0.147

## Data Availability

The datasets used and/or analyzed during the current study are available from the corresponding author upon reasonable request.

## Abbreviations

The following abbreviations are used in this manuscript:

EGFR	Epidermal growth factor receptor
HER	Human Epidermal Growth Factor Receptor
EGFRvIII	Mutated version of Epidermal growth factor receptor
CD44	Cluster differentiation 44
CD109	Cluster differentiation 109
GBM	Glioblastoma
